# Outcomes of the advanced visualization in corneal surgery evaluation trial; a non-inferiority randomized control trial to evaluate the use of intraoperative OCT during Descemet membrane endothelial keratoplasty

**DOI:** 10.3389/fopht.2022.1041778

**Published:** 2023-01-11

**Authors:** Marc B. Muijzer, Heleen Delbeke, Mor M. Dickman, Rudy M.M.A. Nuijts, Herke Jan Noordmans, Saskia M. Imhof, Robert P. L. Wisse

**Affiliations:** ^1^ Utrecht Cornea Research Group, Ophthalmology Department, University Medical Center Utrecht, Utrecht, Netherlands; ^2^ Ophthalmology Department, University Hospital Leuven, Leuven, Belgium; ^3^ Katholieke Universiteit (KU) Leuven, Biomedical Sciences Group, Department of Neurosciences, Research group Ophthalmology, Leuven, Belgium; ^4^ University Eye Clinic, Maastricht University Medical Center, Maastricht, Netherlands; ^5^ Medical Technical and Clinical Physics Department, University Medical Center Utrecht, Utrecht, Netherlands

**Keywords:** intraoperative OCT, iOCT, Descemet membrane endothelial keratoplasty, DMEK, corneal imaging

## Abstract

**Objective:**

To evaluate if an intraoperative-OCT (iOCT) optimized surgical protocol without prolonged overpressure is non-inferior to a standard protocol during Descemet membrane endothelial keratoplasty (DMEK).

**Methods:**

Sixty-five pseudophakic eyes of 65 patients with Fuchs endothelial dystrophy scheduled for routine DMEK were recruited in this prospective non-inferiority international multicenter randomized control trial. Subjects were randomized to the control arm (n=33) without iOCT-use and raising the intraocular pressure above normal physiological limits for 8 minutes (i.e., overpressure) or the intervention arm (n=32) with OCT-guidance to assess graft orientation and adherence, while refraining from prolonged overpressure. The primary outcome was the incidence of postoperative surgery-related adverse events (AE). The non-inferiority margin was set at a risk difference of 10%. Secondary outcomes included iOCT-aided surgical decision making, surgical times, and endothelial cell density (ECD) corrected distance visual acuity (CDVA) at 6 months follow-up.

**Results:**

In the intervention group, 12 subjects developed 13 AEs compared to 13 AEs in 10 subjects in the control group (P=0.644). The risk difference measured -0.32% (95%CI: -10.29 – 9.84). The ECD and CDVA did not differ between the two groups 3 and 6 months postoperatively (P=>0.05). Surgeons reported that iOCT aided surgical decision-making in 40% of cases. Surgery and graft unfolding time were, respectively, 13% and 27% shorter in the iOCT-group.

**Conclusions:**

iOCT-guided DMEK surgery with refraining from prolonged over-pressuring was non-inferior compared to conventional treatment. Surgery times were reduced considerably and iOCT aided surgical decision-making in 40% of cases. Refraining from prolonged overpressure did not affect postoperative ECD or CDVA.

**Clinical trial registration:**

https://clinicaltrials.gov/ct2/show/NCT03763721 (NCT03763721).

## Introduction

Numerous studies described the value of Intraoperative-OCT (iOCT) in ophthalmic surgery (1, 2); iOCT aids in clinical-decision making, enables surgeons to *in-vivo* study their surgical practice patterns, and achieves a greater understanding of pathophysiology and surgical tissue alterations. Nevertheless, most current studies are observational or lacked a control group.

One promising surgery to reap the benefits of iOCT is Descemet membrane endothelial keratoplasty (DMEK) (3–6). DMEK is considered the standard in endothelial keratoplasty (7–9). Despite its advantages, the rate of postoperative adverse events (AEs) for DMEK is relatively high with a reported prevalence of ~20% for rebubbling and ~5% for primary graft failure (9–12). These AEs necessitate secondary surgical interventions and are associated with a lower graft viability and survival (10).

During DMEK surgery the iOCT provides valuable feedback in evaluating graft-host apposition, faster graft positioning with fewer manipulations, and verifying graft orientation in DMEK (3–5). These insights led to the conceptualization of an iOCT-optimized DMEK surgical protocol by our group, consisting of iOCT-guidance during unfolding and refraining from prolonged over-pressuring of the globe. In a pilot study, the incidence of postoperative AEs was lower and operation time was shorter using this protocol (6). Notwithstanding, in this pilot protocol changes were gradually introduced and a control without iOCT guidance was missing. The promising results warranted follow-up in a head-to-head comparison with a conventional surgical protocol. In the current study we prospectively investigate whether iOCT-guidance can obviate the need for prolonged overpressure in DMEK surgery and can be considered non-inferior to a standard protocol in terms of postoperative AEs. Here, we present the results of our prospective *Advanced Visualization In Corneal Surgery Evaluation* (ADVISE), an international non-inferiority randomized clinical trial designed to answer these questions.

## Materials and methods

Subjects underwent routine DMEK surgery between December 2018 and April 2021 in the University Medical Center Utrecht (n = 39), University Hospital Leuven (n = 14), or Maastricht University Medical Center (n= 14). All procedures were performed in accordance with the Declaration of Helsinki, local and national laws regarding research, European directives with respect to privacy, and 2010 CONSORT standards for reporting RCT’s (13) **Supplementary File 3**: Consort Checklist. The study was approved by the Ethics Review Boards in The Netherlands and Belgium (Medical Ethics Committee Utrecht file no. 18-487, Ethical committee Leuven file no. S61527), registered at clinicaltrials.gov (number: NCT03763721) and CCMO.nl (number: NL64392.041.17), and all subjects provided written informed consent.

Inclusion criteria were pseudophakic adult patients with irreversible corneal endothelial dysfunction resulting from Fuchs endothelial corneal dystrophy, eligible for DMEK surgery. Exclusion criteria were human-leukocyte antigen matched keratoplasty, any ocular comorbidity other than ocular surface disease, open angle glaucoma, and mild age-related macular degeneration. No combined phaco-emulsification procedures were performed and only one eye per subject was enrolled. Subjects were randomized to either the iOCT-group or control group using minimization randomization stratified for center using an embedded function of the Electronic Data Capture platform (Research Online, Julius Center, Utrecht, The Netherlands). Patients were blinded throughout the study period. The surgeons and researchers could not be blinded, as the surgeons performed the surgery and researchers were present during surgery to facilitate imaging.

### Study measurements

Each patient underwent a comprehensive ophthalmic examination preoperatively, 3 months, and 6 months after surgery. The ophthalmic examinations included a slit-lamp and fundus examination, intraocular pressure (IOP) measurement, Scheimpflug tomography (Pentacam HR 70900, Oculus GmbH, Wetzlar, Germany), anterior segment OCT (Utrecht and Leuven: Zeiss Cirrus 5000, Zeiss Meditec, Oberkochen, Germany; Maastricht: Casia SS-1000, Tomey, Nagoya, Japan), and posterior segment OCT (Utrecht and Leuven: Zeiss Cirrus 5000, Zeiss Meditec, Oberkochen, Germany; Maastricht: Spectralis, Heidelberg Engineering GmbH, Heidelberg, Germany), and an endothelial cell count using light microscopy (EM4000, Tomey, Nagoya, Japan). Corrected distance visual acuity (CDVA) was measured using an Early Treatment Diabetic Retinopathy Study (ETDRS) letter chart at 4 meters (14). Graft detachments were defined as any non-adherence of the graft noticeable on slit lamp examination and cornea OCT imaging within 6 months after surgery.

### Surgical procedure

Donor grafts were allocated by the Dutch Transplant Foundation (Leiden, the Netherlands). The grafts were organ cultured and provided pre-stripped by ETB-Bislife (Beverwijk, the Netherlands), with a minimum endothelial cell density (ECD) of 2300 cells/mm2 and with a diameter of 8.5 mm. All surgical procedures were performed by experienced corneal surgeons (H.D., R.M.M.A.N, M.M.D., R.P.L.W.), following a largely standardized procedure. Prior to surgery, 27 subjects underwent a Nd : YAG laser iridotomy at 6 o’clock according to the preference of the surgeon. In the other 38 subjects, a surgical iridectomy was performed using a 27-gauge needle and Price hook at 6 o’clock following the Descemetorhexis. In all cases a 2.8 mm corneal incision was made, followed by a 9 mm Descemetorhexis under air in 51 subjects and a viscoelastic device in 14 subjects (Healon; Abbott Medical, Uppsala, Sweden). The graft was stained using trypan blue dye (Membrane blue n = 52, Vision Blue, n=13, both from DORC, Zuidland, the Netherlands) and inserted into the anterior chamber using a glass injector. A “no touch technique” was used to unfold and position the graft (15). In 33 surgeries the randomization dictated that iOCT was not available to the surgeons. Here, a full AC fill was performed, raising the IOP above normal physiological limits for 8 minutes using air (*overpressure)*. In the other 32 surgeries the graft was positioned as described above, the iOCT (Lumera 700 OPMI Rescan, Zeiss Meditec, Oberkochen, Germany) was available for utilization at the surgeon’s discretion during unfolding and used to check for complete adherence of the graft without overpressurizing the eye. In both groups at the end of surgery, a the air was replaced by 20% Sulphur Hexafluoride gas and the size of the gas bubble was reduced to cover the graft (i.e., same size as the graft). Next, a validation scan of proper apposition by iOCT was performed in both the control and intervention arm, as proposed by the ethical review board. Any irregularities were treated at the discretion of the surgeon. After surgery, patients remained strictly supine for two hours in the hospital and were instructed to remain supine for the following 24 hours. Directly after the surgery the surgeons were asked on whether the iOCT-aided surgical decision making and if applicable how the iOCT-aided surgical decision making.

All surgical videos were qualitatively analyzed by two graders to record graft unfolding grade and surgical times. Graft unfolding difficulty was classified in 4 grades depending on the required manipulation and time to unfold/position the graft as earlier described by Maier et al. (16)

### Outcome measures

The primary outcome was the incidence of postoperative AEs, defined as graft detachments requiring surgical intervention (i.e., rebubbling), primary graft failures, or iatrogenic acute glaucoma. Rebubbling was performed at the discretion of the surgeon, though principally when the graft was >30% detached or the detachment involved the visual axis. Secondary outcomes consisted of surgeon reported iOCT-aided surgical decision-making, surgical time, postoperative ECD loss, and CDVA at follow-up.

### Sample size

Power calculation was based on the incidence of postoperative AEs. Assuming an α of 0.05 (1-sided) and a power of 80%, and a non-inferiority limit of 10%, a sample size of at least 60 subjects would be required (30 per treatment arm). Considering a loss to follow up of 5%, the final computed sample size was 63 subjects. The power calculation did not provide for COVID-19 related loss to follow-up (n = 4).

### Statistical analysis

The primary outcome consisted of the total counted AEs developed by each patient and converted to a proportion for analysis. A crude and adjusted marginal risk difference (RD) between the two treatment arms was estimated from a logistic regression model using 1,000 bootstrap samples and adjusted for treatment site (17). *P*-values cannot be calculated from the described method and only can be estimated using the 95% CI. The analysis was stratified to calculate the unadjusted RD for graft detachment, rebubbling, primary graft failure, and iatrogenic acute glaucoma. For a stratified adjusted analysis, it appeared not possible to calculate reliable estimates, because the study was not powered to compare the separate adverse events. In addition, a regression analysis was performed to estimate the effect of overpressure duration in minutes on the incidence of graft detachment and area of detachment. All secondary outcomes were analyzed for differences between treatment arms using the student t-test or Fisher-exact test as appropriate. Correction for multiple comparisons was performed using the Bonferroni correction. A 2-sided *P* value < 0.05 was considered statistically significant.

An intention-to-treat analysis was performed for all outcomes measures. All statistical analysis were performed using R statistical software version 4.0.3 (CRAN, Vienna, Austria) and supervised by an independent statistician from the Julius Center for Health Sciences. In February 2020 an interim analysis and data and safety monitoring board evaluation was performed, recommending to proceed with the study without changes. Missing observation of the secondary outcomes were imputed using multiple imputation. Missing measurements of subjects that developed a graft failure were considered missing not at random and not imputed.

## Results

**Figure 1:**
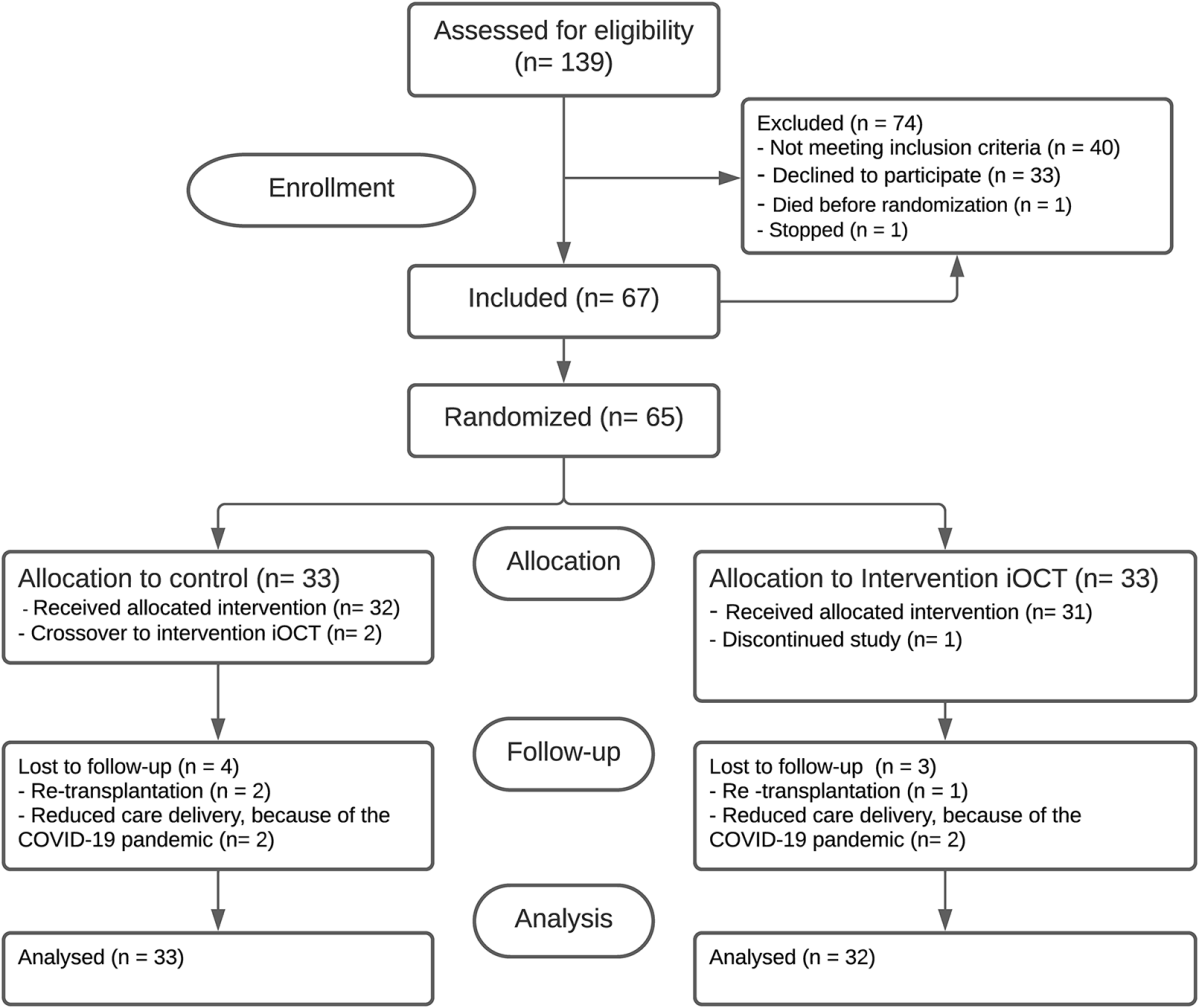
Consolidated standards of reporting trials flowchart.

A total of 65 eyes of 65 patients received either the conventional protocol (control group, n = 33) or the iOCT-optimized protocol (intervention group, n = 32). In the control group 2 cross-over cases were recorded, in which the iOCT was used to salvage the graft in a complicated procedure. In both cases 8 minutes of overpressure was applied at the end of surgery. All remaining patients in both treatment arms received the allocated treatment. Four serious adverse events (SAE) were recorded over the course of the study; 3 subjects underwent re-transplantation for primary graft failure and one subject died of multi-organ failure unrelated to the study before randomization and was excluded without replacement. In total, 7 subjects were lost to follow-up; 3 subjects dropped out after re-transplantation and 4 subjects were lost to follow-up because of reduction in care delivery caused by the COVID-19 pandemic. For all subjects the primary outcome was obtained and included for analysis (**Figure 1**). Baseline patient and donor characteristics are displayed in **Table 1**.

**Table 1:** Baseline patient and donor characteristics.

	Conventional protocol (n=33)	iOCT-optimized protocol (n= 32)
Patient		
Sex (female), n (%)	17 (52)	17 (53)
Age (years), mean (SD)	72.4 (6.6)	73.3 (6.4)
CDVA (logMAR), mean (SD)	0.42 (0.25)	0.41 (0.26)
Pachymetry (µm), mean (SD)	625 (86)	595 (62)
RFNL thickness (µm), mean (SD)	89 (13)	87 (13)
IOP (mmHg), mean (SD)	12.9 (3.3)	12.6 (3.0)
Corneal edema present, n (%)	15 (45.5)	13 (40.6)
Descemet folds present, n (%)	2 (6.1)	6 (18.8)
Bullae present, n (%)	5 (15.2)	4 (12.5)
Laser iridotomy, n (%)	15 (45.5)	12 (37.5)
Donor		
Age (years), mean (SD)	74.3 (5.0)	73.3 (5.8)
ECD (cells/mm^2^), mean (SD)	2706 (174)	2719 (180)

CDVA, corrected distance visual acuity; ECD, endothelial cell density; logMAR, logarithm of the minimum angle of resolution; IOP, intra-ocular pressure; SD, standard deviation; RFNL, retinal nerve fiber layer.

Commensurate with the 2012 CONSORT guidelines, baseline characteristics were not tested for statistical differences (13).

### Incidence of postoperative adverse events and clinical outcomes

A total of 26 postoperative AEs were recorded in 22 subjects (control group: 13 AEs in 10 subjects, intervention group: 13 AEs in 12 subjects). No statistically significant differences in the incidence of AEs were found between the intervention group and the control group (**Table 2**).

**Table 2:** Primary and secondary outcomes after conventional treatment and iOCT-optimized treatment.

		Conventional treatment (control group, n=33)	iOCT-optimized treatment (n = 32)	P-value^a^ (adj^b^)
CDVA (LogMAR), mean (SD)				
	3 months	0.14 (0.13)	0.18 (0.19)	0.342 (0.684)
	6 months	0.13 (0.14)	0.22 (0.29)	0.138 (0.276)
Pachymetry (µm), mean (SD)				
	3 months	478.33 (40.69)	470.88 (51.54)	0.519 (1.000)
	6 months	486.79 (52.13)	487.16 (55.57)	0.978 (1.000)
ECD (cells/mm^2^), mean (SD)				
	3 months	1852.81 (375.06)	1756.35 (414.97)	0.341 (0.682)
	6 months	1838.06 (359.84)	1708.81 (479.70)	0.235 (0.470)
ECD loss (cells/mm^2^), mean (SD)				
	3 months	838.50 (377.48)	963.00 (393.50)	0.213 (0.426)
	6 months	857.37 (334.89)	1010.55 (450.25)	0.138 (0.276)
RFNL thickness (µm), mean (SD)				
	3 months	91.15 (13.31)	90.78 (12.93)	0.910 (1.000)
	6 months	89.85 (12.42)	90.38 (14.51)	0.876 (1.000)
IOP (mmHg), mean (SD)				
	3 months	15.03 (2.98)	15.09 (4.29)	0.945 (1.000)
	6 months	14.36 (3.85)	15.19 (5.15)	0.467 (0.934)
Total adverse events, n (%)^c^		13 (39.4)	13 (40.6)	0.644 (1.00)
Detachments		16 (48.5)	17 (53.1)	0.900 (1.00)
Rebubbling		6 (18.2)	11 (34.4)	0.229 (1.00)
Graft failure		2 (6.1)	1 (3.1)	1.000 (1.00)
Iatrogenic acute glaucoma		5 (15.2)	1 (3.1)	0.213 (1.00)

CDVA, corrected distance visual acuity; ECD, endothelial cell density; logMAR, logarithm of the minimum angle of resolution; IOP, intra-ocular pressure; iOCT, intraoperative optical coherence tomography; SD, standard deviation; RFNL, retinal nerve fiber layer.

a Independent samples Student´s t-test.

b Adjusted for multiple comparisons using Bonferroni method.

c Summation of the primary outcomes, defined as rebubbling, graft failure and iatrogenic glaucoma.

**Figure 2:**
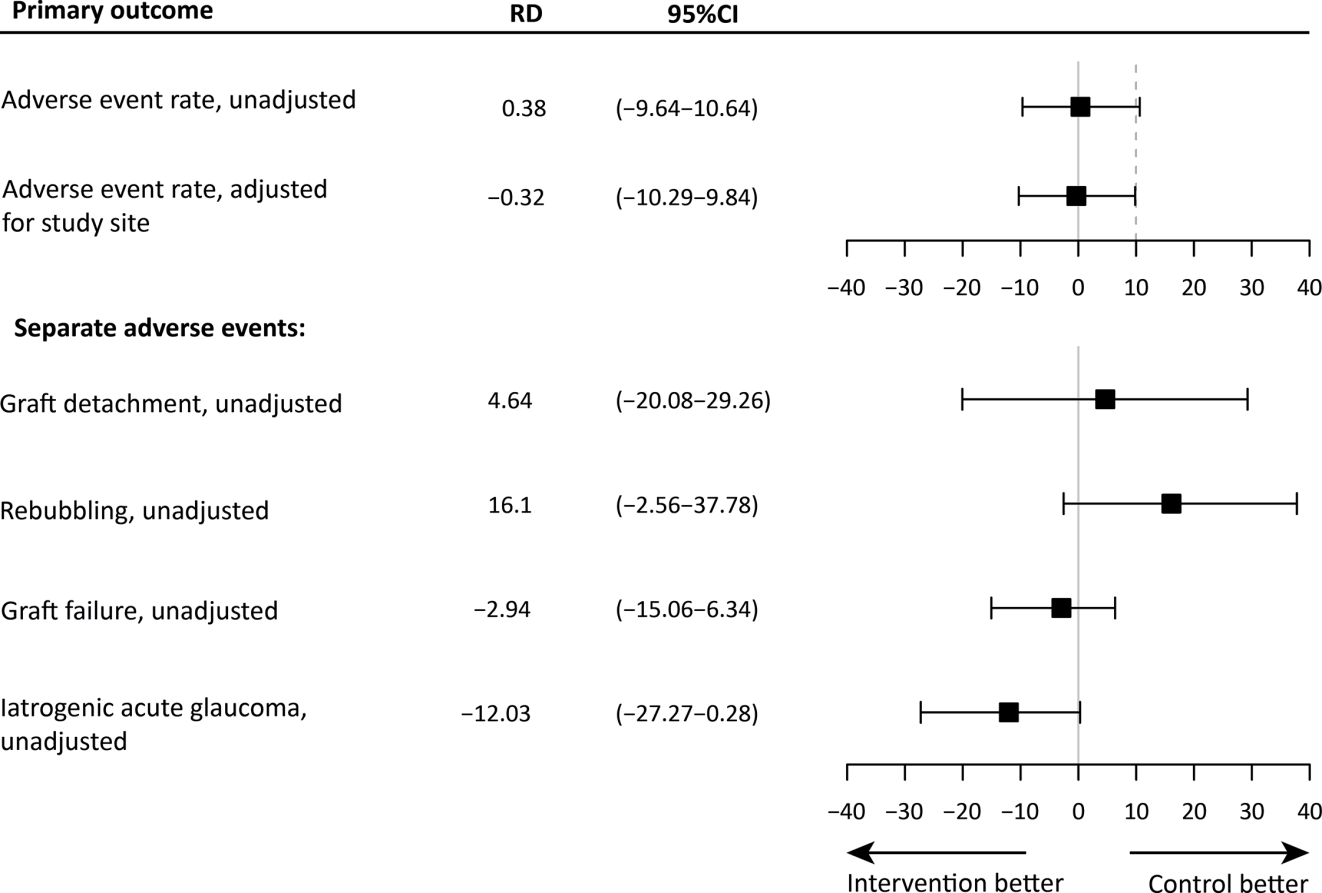
The mean risk difference (RD) and 95% confidence interval (CI) of the outcome measures, and the non-inferiority limit (dashed line). The top panel shows the unadjusted and adjusted estimates for the primary outcome measure. The bottom panel shows the unadjusted estimates for all separate postoperative events. For these outcomes, a non-inferiority margin is not shown.

The mean unadjusted risk difference (RD) was 0.38% (95%CI: -9.64 – 10.64) and the RD adjusted for study site was -0.32% (95%CI: -10.29 – 9.84), meaning that both protocols are comparable with regards to overall surgical safety measured as total postoperative AE rate (**Figure 2**; **Supplementary Table 1**). After controlling for *a priori* planned adjustment for study site, the iOCT-optimized protocol was found non-inferior to the conventional protocol. **Figure 2**, also report on the individual AEs, where the analysis shows a high uncertainty regarding their effect sizes as the trial was not designed to assess these individual adverse events. In addition, the independent effect of overpressure duration measured in minutes was not significantly associated with the incidence of detachment (95%CI: -0.10–0.15, *P* = 0.730) or area of detachment (95%CI: -0.027 – 0.002, *P* = 0.121)

No significant differences were found between the control group and the iOCT group regarding secondary clinical outcomes at 3 and 6 months postoperative (**Table 2**).

### Surgical decision-making and surgical duration

In 35 surgeries the iOCT was utilized. The use of iOCT salvaged the graft in one cross-over case. The other cross-over graft was correctly positioned though eventually developed an early graft failure, presumably due to excess manipulation. None of the iOCT-group cases exhibited interface irregularities or graft detachment at the end of surgery. The obligatory verification scan in the control group revealed peripheral detachment of the graft in one case, resulting in repositioning of the graft and subsequent overpressure for another 4 minutes. Notwithstanding, this case developed a detachment for which a rebubbling was performed.

Surgeons reported that the iOCT benefited decision-making in 14 of 35 cases (40%); in all cases iOCT aided determining graft orientation (incl. 8 grafts inserted upside-down) and in 3 cases iOCT aided unfolding and positioning of the graft. The median time the iOCT was used measured 2 minutes and 52 seconds (IQR: 03:43, range: 00:19 – 23:40). Graft unfolding difficulty was significantly associated with iOCT-aided surgical decision-making (P = 0.011, **Supplementary Table 2**), graft unfolding difficulty did not differ between both treatment arm (P = 0.474, **Supplementary Table 2**).

The mean surgical skin-to-skin time and graft unfolding time were, respectively, 13% and 27% shorter compared to the control group (**Table 3**).

**Table 3:** Overview of surgical times Manually scored and video-graded by two independent observers.

	Conventional treatment (control group)	iOCT-optimized treatment	Relative difference (%)	95% CI
Surgical skin-to-skin time (minutes), mean (SD)	37.62 (10.09)	32.72 (10.99)	-13.0	-0.36 – 10.18
Overpressure time(minutes), mean (SD)	9.73 (1.94)	2.73 (1.29)	-71.9	6.19 – 7.82
unfolding time, minutes, (minutes), mean (SD)	6.26 (8.13)	4.58 (5.35)	-26.8	-1.75 – 5.10
Graft preparation time^a^, (minutes), mean (SD)	6.83 (1.95)	6.97 (2.01)	2.0	-1.25 – 0.98
Descemetorhexis time, (minutes), mean (SD)	4.35 (4.41)	5.90 (4.84)	35.6	-3.88 – 0.78

SD, standard deviation; iOCT, intraoperative optical coherence tomography.

a Grafts preparation time for surgeries in Leuven was not available.

## Discussion

In this study we found that an iOCT-optimized DMEK surgical protocol refraining from over pressurizing was non-inferior compared to a conventional protocol, with no iOCT-guidance and standard 8 minutes of overpressure. Our results do not support the perceived benefit of overpressure to promote graft adherence. Though the independent effect of iOCT use on surgical safety could not be reliably estimated, the benefits of our iOCT-optimized protocol are a shorter surgical skin-to-skin time (-13%) and assisted surgical decision making (40% of cases). Furthermore, the access to iOCT and its improved visualization proved crucial during surgery in 9% of cases in the control group (2 crossovers and 1 validation scan with observed intra-operative graft detachment).

The causes of graft detachments are considered multifactorial and a large body of research reported on risk factors, such as donor and recipient characteristics, and intraoperative factors such as overpressure of the globe (10, 18–20). Over-pressuring during surgery is considered by some as a protective factor against graft detachments (21, 22), whereas two cohort studies did not support this (23, 24). Our study is the first head-to-head comparison of over-pressuring in DMEK surgery, and our data do not support the notion that over-pressure prevents graft detachments nor rebubbling procedures.

Overall, the prevalence of AEs did not differ materially between both treatment arms, though the nature of the separate AEs differed, such as less iatrogenic acute glaucoma in the eyes without overpressure. Interestingly, graft detachments occurred at an equal rate (n=17 vs. n=16) and the areas of detachment were of comparable size (detached area: 44% versus 39%), though rebubbling procedures were performed more often in the iOCT group. The cause of this difference remain unclear as our study was not designed to assess nor explore predictors for clinical decision making regarding rebubbling procedures.

The use of iOCT benefitted the surgical decision-making process in 40% of cases. This finding is consistent with results from comparable studies, including our pilot study and the landmark PIONEER and DISCOVER studies (3–6, 25, 26). Similar to these studies, our surgeons reported that the iOCT imaging was particularly advantageous for assessing graft orientation and in lesser degree during the unfolding of the graft. Interestingly, we found a significant association between reported iOCT-aided surgical decision-making and the graded unfolding difficulty. This makes sense, as the circumstances and causes which make graft orientation difficult to assess (e.g., poor visualization, graft geometry and tissue properties) may also increase the difficulty of unfolding the graft (16)

The surgical skin-to-skin time was 13% shorter in the iOCT group, which was expected due to refraining from overpressure. In line with similar reports, we found that the iOCT enables the surgeon in a 27% faster unfolding and positioning of the graft. Though not assessed in this study a shorter duration of unfolding and positioning the graft may be related to less manipulation of the graft and improved graft viability and survival (3–5). Efficiency gains from refraining from overpressure and a faster unfolding time may be offset by the time taken to assess the iOCT images. Future development in automated image analysis may aid to reduce this offset (1, 27, 28).

Long-term follow-up results appeared comparable for both groups. The postoperative ECD loss was slightly lower in the iOCT-optimized protocol compared with the conventional protocol, albeit not statistically significant. In addition to other reports, the combined results suggest that ECD, postoperative IOP or retinal nerve fiber layer are not affected by prolonged overpressure (22, 24, 29).

We evaluated the use of DMEK and use of overpressure. However, the partial effect of these individual factors is difficult to estimate reliably due to the introduced multi-collinearity. In selected cases, iOCT proved indispensable for the surgeon to complete the surgery successfully, and many reports highlight this benefit of iOCT, though it is not feasible to power a trial on these rare cases and outcomes (1, 30). Additionally, when iOCT is available at a center, withholding this technology is considered unethical. We firmly believe that new innovations should be tested on endpoints relevant for patients, and assessing process-related outcomes (e.g. surgical time) can only be secondary to a primary outcome that relates directly to the patient (e.g. surgical safety). With this the relative costs of the iOCT-system warrant discussion given the non-inferiority of our iOCT-optimized protocol. The advantages of iOCT (e.g., time, decision-making) were not reflected in overall post-operative AE rates and clinical outcomes. Similarly, one could hypothesize that refraining from overpressure is also non-inferior regardless intraoperative imaging, and this should be confirmed by clinical studies. Another consideration is in the interpretation of outcomes regarding graft detachments and rebubbling events. In our study protocol, we listed rebubbling as a primary outcome due to its relevance from a patient perspective, though advancing insights let to the conclusion that a graft detachment is a more objective and quantifiable outcome. We therefore reported both and acknowledge that the decision to re-adhere a graft is made by the surgeon.

In conclusion, iOCT-guided DMEK surgery refraining from prolonged over-pressurizing was proven non-inferior to a conventional approach, though it did not reduce the overall rate of post-operative AEs. Surgery times were reduced overall by 13% and the iOCT resulted in a 27% reduction of unfolding time. Surgeons reported a benefit of iOCT in 40% of cases, and iOCT was indispensable in 9% of the conventional cases.

## Data availability statement

The raw data supporting the conclusions of this article will be made available by the authors, without undue reservation.

## Ethics statement

The studies involving human participants were reviewed and approved by Medisch Ethische toetsingscommissie Utrecht. The patients/participants provided their written informed consent to participate in this study.

## Author contributions

MM had full access to all the data in the study and take responsibility for the integrity of the data and the accuracy of the data analysis. Concept and design: All authors. Acquisition, analysis, or interpretation of data: All authors. Drafting of the manuscript MM, RW. Critical revision of the manuscript for important intellectual content: All authors. Statistical analysis: MM. Obtained funding: RW. Administrative, technical, or material support: RW, SI, HN. Supervision: RW, SI, HN. All authors contributed to the article and approved the submitted version.

## References

[1] Muijzer MB Schellekens PAWJ Beckers HJM de Boer JH Imhof SM Wisse RPL. Clinical applications for intraoperative optical coherence tomography: A systematic review. Eye (2021) :1–13. doi: 10.1038/s41433-021-01686-9 PMC8807841 34272509

[2] Posarelli C Sartini F Casini G Passani A Toro MD Vella G. What is the impact of intraoperative microscope-integrated OCT in ophthalmic surgery? relevant applications and outcomes. a systematic review. J Clin Med (2020) 9(6):1682. doi: 10.3390/jcm9061682 32498222 PMC7356858

[3] Saad A Guilbert E Grise-Dulac A Sabatier P Gatinel D. Intraoperative OCT-assisted DMEK: 14 consecutive cases. Cornea (2015) 34(7):802–7. doi: 10.1097/ICO.0000000000000462 26002152

[4] Patel AS Goshe JM Srivastava SK Ehlers JP. Intraoperative optical coherence tomography–assisted descemet membrane endothelial keratoplasty in the DISCOVER study: First 100 cases. Am J Ophthalmol (2020) 210(February 2018):167–73. doi: 10.1016/j.ajo.2019.09.018 PMC7002191 31562854

[5] Cost B Goshe JM Srivastava S Ehlers JP. Intraoperative optical coherence tomography-assisted descemet membrane endothelial keratoplasty in the DISCOVER study. Am J Ophthalmol (2015) 160(3):430–7. doi: 10.1016/j.ajo.2015.05.020 PMC4544647 26026264

[6] Muijzer MB Soeters N Godefrooij DA van Luijk CM Wisse RPL. Intraoperative optical coherence tomography-assisted descemet membrane endothelial keratoplasty: Toward more efficient, safer surgery. Cornea (2020) 39(6):674–9. doi: 10.1097/ICO.0000000000002301 32141944

[7] Dunker SL Dickman MM Wisse RPL Nobacht S Wijdh RHJ Bartels MC. Descemet membrane endothelial keratoplasty versus ultrathin descemet stripping automated endothelial keratoplasty: A multicenter randomized controlled clinical trial. Ophthalmology (2020) 127(9):1152–9. doi: 10.1016/j.ophtha.2020.02.029 32386811

[8] Dunker SL Veldman MHJ Winkens B van den Biggelaar FJHM Nuijts RMMA Kruit PJ. Real-world outcomes of DMEK: A prospective Dutch registry study. Am J Ophthalmol (2021) 222:218–25. doi: 10.1016/j.ajo.2020.06.023 32621899

[9] Stuart AJ Romano V Virgili G Shortt AJ. Descemet’s membrane endothelial keratoplasty (DMEK) versus descemet’s stripping automated endothelial keratoplasty (DSAEK) for corneal endothelial failure. Cochrane Database Syst Rev. (2018) 6(6):CD012097. doi: 10.1002/14651858.CD012097.pub2 29940078 PMC6513431

[10] Dunker S Winkens B Van Den Biggelaar F Nuijts R Kruit PJ Dickman M. Rebubbling and graft failure in descemet membrane endothelial keratoplasty: A prospective Dutch registry study. Br J Ophthalmol (2021) :1–7. doi: 10.1136/bjophthalmol-2020-317041 PMC9763158 33597192

[11] Singh A Zarei-Ghanavati M Avadhanam V Liu C. Systematic review and meta-analysis of clinical outcomes of descemet membrane endothelial keratoplasty versus descemet stripping endothelial Keratoplasty/Descemet stripping automated endothelial keratoplasty. Cornea (2017) 36(11):1437–43. doi: 10.1097/ICO.0000000000001320 28834814

[12] Li S Liu L Wang W Huang T Zhong X Yuan J. Efficacy and safety of descemet’s membrane endothelial keratoplasty versus descemet’s stripping endothelial keratoplasty: A systematic review and meta-analysis. PloS One (2017) 12(12):1–21. doi: 10.1371/journal.pone.0182275 PMC5734733 29252983

[13] Schulz KF Altman DG Moher D. CONSORT 2010 statement: Updated guidelines for reporting parallel group randomised trials. BMJ (2010) 340:c332. doi: 10.1136/bmj.c332 20332509 PMC2844940

[14] Beck RW Moke PS Turpin AH Ferris FL SanGiovanni JP Johnson CA. A computerized method of visual acuity testing: Adaptation of the early treatment of diabetic retinopathy study testing protocol. Am J Ophthalmol (2003) 135(2):194–205. doi: 10.1016/S0002-9394(02)01825-1 12566024

[15] Dapena I Moutsouris K Droutsas K Ham L van Dijk K Melles G. Standardized “No-touch” technique for descemet membrane endothelial keratoplasty. Arch Ophthalmol (2011) 129(1):88–94. doi: 10.1001/archophthalmol.2010.334 21220634

[16] Maier AKB Gundlach E Schroeter J Klamann MKJ Gonnermann J Riechardt AI. Influence of the difficulty of graft unfolding and attachment on the outcome in descemet membrane endothelial keratoplasty. Graefe’s Arch Clin Exp Ophthalmol (2015) 253(6):895–900. doi: 10.1007/s00417-015-2939-9 25631845

[17] Austin PC. Absolute risk reductions, relative risks, relative risk reductions, and numbers needed to treat can be obtained from a logistic regression model. J Clin Epidemiol (2010) 63(1):2–6. doi: 10.1016/j.jclinepi.2008.11.004 19230611

[18] Parekh M Leon P Ruzza A Borroni D Ferrari S Ponzin D. Graft detachment and rebubbling rate in descemet membrane endothelial keratoplasty. Surv Ophthalmol (2018) 63(2):245–50. doi: 10.1016/j.survophthal.2017.07.003 28739402

[19] Weller JM Schlötzer-Schrehardt U Tourtas T Kruse FE. Influence of ultrastructural corneal graft abnormalities on the outcome of descemet membrane endothelial keratoplasty. Am J Ophthalmol (2016) 169:58–67. doi: 10.1016/j.ajo.2016.06.013 27318075

[20] Quilendrino R Rodriguez-Calvo de Mora M Baydoun L Ham L van Dijk K Dapena I. Prevention and management of graft detachment in descemet membrane endothelial keratoplasty. Arch Ophthalmol (2012) 130(3):280–91. doi: 10.1001/archophthalmol.2011.343 22084160

[21] Terry MA Straiko MD Veldman PB Talajic JC VanZyl C Sales CS. Standardized DMEK technique: Reducing complications using prestripped tissue, novel glass injector, and sulfur hexafluoride (SF6) gas. Cornea (2015) 34(8):845–52. doi: 10.1097/ICO.0000000000000479 26075461

[22] Fajgenbaum MAP Hollick EJ. Does same-day postoperative increased intraocular pressure affect endothelial cell density after descemet membrane endothelial keratoplasty? Cornea (2018) 37(12):1484–9. doi: 10.1097/ICO.0000000000001762 30222712

[23] Schmeckenbächer N Frings A Kruse FE Tourtas TL. Role of initial intraocular pressure in graft adhesion after descemet membrane endothelial keratoplasty. Cornea (2017) 36(1):7–10. doi: 10.1097/ICO.0000000000001055 27755190

[24] Pilger D Wilkemeyer I Schroeter J Maier AKB Torun N. Rebubbling in descemet membrane endothelial keratoplasty: Influence of pressure and duration of the intracameral air tamponade. Am J Ophthalmol (2017) 178:122–8. doi: 10.1016/j.ajo.2017.03.021 28342720

[25] Ehlers JP Dupps WJ Kaiser PK Goshe J Singh RP Petkovsek D. The prospective intraoperative and perioperative ophthalmic ImagiNg with optical CoherEncE TomogRaphy (PIONEER) study: 2-year results. Am J Ophthalmol (2014) 158(5):999–1007. doi: 10.1016/j.ajo.2014.07.034 25077834 PMC4250395

[26] Ehlers JP Modi YS Pecen PE Goshe J Dupps WJ Rachitskaya A. The DISCOVER study 3-year results: Feasibility and usefulness of microscope-integrated intraoperative OCT during ophthalmic surgery. Ophthalmology (2018) 125(7):1014–27. doi: 10.1016/j.ophtha.2017.12.037 PMC6015779 29409662

[27] Xu D Dupps WJJ Srivastava SK Ehlers JP. Automated volumetric analysis of interface fluid in descemet stripping automated endothelial keratoplasty using intraoperative optical coherence tomography. Invest Ophthalmol Vis Sci (2014) 55(9):5610–5. doi: 10.1167/iovs.14-14346 PMC4160074 25103262

[28] Muijzer MB Heslinga FG Couwenberg F Noordmans H-J Oahalou A Pluim JPW. Automatic evaluation of graft orientation during descemet membrane endothelial keratoplasty using intraoperative OCT. BioMed Opt Express (2022) 13(5):2683–94. doi: 10.1364/BOE.446519 PMC9203112 35774322

[29] Fortune B Yang H Strouthidis NG Cull GA Grimm JL Downs JC. The effect of acute intraocular pressure elevation on peripapillary retinal thickness, retinal nerve fiber layer thickness, and retardance. Investig Ophthalmol Vis Sci (2009) 50(10):4719–26. doi: 10.1167/iovs.08-3289 PMC2764538 19420342

[30] Muijzer MB Kroes HY van Hasselt PM Wisse RPL. Bilateral posterior lamellar corneal transplant surgery in an infant of 17 weeks old: Surgical challenges and the added value of intraoperative optical coherence tomography. Clin Case Rep (2022) 10:e05637. doi: 10.1002/ccr3.5637 35387289 PMC8978779

