# Supplementary material for: Outcomes of the advanced visualization in corneal surgery evaluation trial; a non-inferiority randomized control trial to evaluate the use of intraoperative OCT during Descemet membrane endothelial keratoplasty
Source: Front Ophthalmol (Lausanne). 2023 Jan 11;2:1041778. doi: 10.3389/fopht.2022.1041778 (PMC11182175; doi:10.3389/fopht.2022.1041778)
Supplement: Supplementary file 1 [file Table_1.docx]

Supplementary table 1. Logistic regression models for incidence of total adverse event rate

|  | **Unadjusted model** | | **Adjusted model** | |
| --- | --- | --- | --- | --- |
| **Variables** | **OR (95%CI)** | **P** | **OR (95%CI)** | **P** |
| iOCT-optimized protocol | 1.036  (0.454 - 2.366) | 0.933 | 0.976  (0.421 – 2.263) | 0.955 |
| Study site 2^1^ | - | - | 0.123  (0.016 – 0.953) | 0.045 |
| Study site 3^1^ | - | - | 0.634  (0.221 – 1.819) | 0.396 |

^1 Reference: study site 1
iOCT: intraoperative optical coherence tomography^
