# Supplementary material for: Outcomes of the advanced visualization in corneal surgery evaluation trial; a non-inferiority randomized control trial to evaluate the use of intraoperative OCT during Descemet membrane endothelial keratoplasty
Source: Front Ophthalmol (Lausanne). 2023 Jan 11;2:1041778. doi: 10.3389/fopht.2022.1041778 (PMC11182175; doi:10.3389/fopht.2022.1041778)
Supplement: Supplementary file 2 [file Table_2.docx]

Supplementary table 2. Overview of graft unfolding grade in both treatment arms

| **Treatment arm / Graft unfolding grade^1^** | | **I** | **II** | **III** | **IV** | **P-value**^2^ |
| --- | --- | --- | --- | --- | --- | --- |
| Conventional protocol, **n** | | 4 | 14 | 4 | 11 | 0.474 |
| iOCT-optimized protocol, **n** | | 7 | 14 | 1 | 9 |  |
|  | iOCT aided surgical decision-making | 1 | 4 | 1 | 7 | 0.011 |
|  | iOCT did not aid surgical decision-making | 6 | 10 | 0 | 2 |  |

**^1^** Graft unfolding grade is classified in 4 grades depending on the required manipulation and time to unfold/position the graft. Grade I: graft lamella primarily oriented correctly in the anterior chamber, straight and direct unfolding and centering; Grade II: slightly complicated, indirect unfolding and centering (duration less than five min); Grade III: difficult indirect unfolding and centering (duration longer than five min), repeated air injection with BSS exchange necessary; Grade IV: direct manipulation of the graft lamella for unfolding and centering by cannula or forceps.
**^2^** Fisher exact test
